# The cost-effectiveness of improved brief interventions for tobacco cessation in Thailand

**DOI:** 10.3389/fpubh.2023.1289561

**Published:** 2023-11-23

**Authors:** Rungrat Palakai, Bundit Sornpaisarn, Yothin Sawangdee, Sutthida Chuanwan, Pairoj Saonuam, Piyawat Katewongsa, Jürgen Rehm

**Affiliations:** ^1^Institute for Population and Social Research, Mahidol University, Nakhon Pathom, Thailand; ^2^Institute for Mental Health Policy Research, Centre for Addiction and Mental Health (CAMH), Toronto, ON, Canada; ^3^Dalla Lana School of Public Health, University of Toronto, Toronto, ON, Canada; ^4^Faculty of Public Health, Mahidol University, Bangkok, Thailand; ^5^Healthy Lifestyle Promotion Section, Thai Health Promotion Foundation, Bangkok, Thailand; ^6^Thailand Physical Activity Knowledge Development Centre (TPAK), Institute for Population and Social Research, Mahidol University, Nakhon Pathom, Thailand; ^7^Campbell Family Mental Health Research Institute, CAMH, Toronto, ON, Canada; ^8^Department of Psychiatry, University of Toronto, Toronto, ON, Canada; ^9^Institute of Medical Science, University of Toronto, Toronto, ON, Canada; ^10^Department of International Health Projects, Institute for Leadership and Health Management, I.M. Sechenov First Moscow State Medical University, Moscow, Russia

**Keywords:** tobacco cessation, economic evaluation, cost-effectiveness analysis, brief intervention (BI), Thailand

## Abstract

**Background:**

This study estimated the cost-effectiveness of four strategies enhancing the quality and accessibility of Brief Intervention (BI) service for smoking cessation in Thailand during 2022–2030: (1) current-BI (status quo), (2) the effective-training standard-BI, (3) the current-BI plus the village health volunteers (VHV) mobilization, and (4) the effective-training BI plus VHV mobilization.

**Methods:**

By interviewing five public health officers, nine healthcare professionals aiding these services, and fifteen BI service experts, we explored the status quo situation of the Thai smoking cessation service system, including main activities, their quantity assumptions, and activities’ unit prices needed to operate the current cessation service system. Then, we modeled additional activities needed to implement the other three simulated scenarios. We estimated the costs and impacts of implementing these strategies over a nine-year operating horizon (2022–2030), covering 3 years of service system preparation and 6 years of full implementation. The modeled costs of these four strategies included intervention and program costs. The study focused on current smokers age 15 years or older. The assessed impact parameters encompassed smoking prevalence, deaths averted, and healthy life-years gained. An Incremental Cost-Effectiveness Analysis compared the four simulated strategies was employed. Data analysis was performed using the One Health Tool software, which the World Health Organization developed.

**Results:**

The findings of this investigation reveal that all three intervention strategies exhibited cost-effectiveness compared to the prevailing status quo. Among these strategies, Strategy 2, enhancing BI service quality, emerged as the most efficient and efficacious option. Therefore, the expansion of quality services should be synergistically aligned with augmented training, service delivery optimization, and managerial enhancements.

**Conclusion:**

This approach is particularly poised to enhance accessibility to and the efficacy of smoking cessation interventions across Thailand.

## 1 Introduction

Causes of death globally have evolved epidemiologically from infectious diseases to chronic degenerative diseases. In 2020, noncommunicable diseases (NCD) were responsible for 41 million deaths, which include eight million deaths from smoking (1, 2). For this reason, WHO has developed a global action plan for NCD prevention and control. One of the global targets is to reduce smoking rates by 30% between 2010 and 2025. The reduction of tobacco and other risk factors is necessary to achieve the Sustainable Development Goal (SDG) of reducing the number of premature deaths from NCD by 2030 (3, 4). In Thailand, smoking prevalence began to decline in 1991 from a peak of 32.0% down to 20.7% in 2009. Prevalence of smokers remained constant at around 19–20% until 2017 (5). The 2021 National Statistical Office’s (NSO) Report on Tobacco Consumption Behavior survey showed that the prevalence of smoking in the Thai population age 15 years or older was 17.4%, with a prevalence of 34.7% among males and 1.3% among females (6). According to the 2015 National Health Survey in Thailand, two-thirds of smokers wanted to quit smoking, and 55% had tried, but only 7% were successful (7).

To effectively control tobacco consumption, WHO has developed the Framework Convention on Tobacco Control (FCTC), which has 168 signatory countries (8). The FCTC is operationalized through the MPOWER policy package. The MPOWER acronym stands for: (i) Monitoring tobacco uses and prevention measures, (ii) Protecting people from tobacco use, (iii) Offering cessation assistance, (iv) Warning about the dangers of tobacco, (v) Enforcing bans on tobacco advertising, and (vi) Raising taxes on tobacco (9). MPOWER has been shown to be effective in reducing tobacco use, shown by a reduction in the lung cancer mortality rate from 7 to 5% in Australia (10).

The “O” component of MPOWER refers to offering individual behavioral counseling, i.e., a brief intervention (BI) to help smokers quit smoking. For smoking cessation, the BI method consists of up to four face-to-face counseling sessions, with 5–10 min for each counseling session (11–13). The BI method increased the smoking cessation rate by an average of two percentage points of all current smokers within 1 year in the U.S. (14). In addition, a systematic review of 13 primary studies found that the BI achieved a success rate of 40% for quit attempts for its recipients (15, 16). This study estimated the cost-effectiveness of various hypothetical strategies aimed at enhancing the quality and accessibility of Brief Intervention (BI) service for smoking cessation in Thailand.

As mentioned earlier, BI has been shown to help smokers quit and, thus, the Thai health system has offered the BI service for smokers free of charge (17). In 2021, primary care providers verbally screened 15,281,599 people (28.8% of Thais age 15 years or over), of whom 9,921,801 said they were smokers. Providers offered smokers who wanted to quit help with quitting. However, only 1,548,287 smokers (15.6%) received help to quit tobacco use, and only 57,172 smokers (3.7%) who received BI successfully quit smoking (18). Those findings reflect two shortcomings of the Thai BI service system for smoking cessation: low access and poor efficacy.

In pursuit of enhancing the efficacy of Thailand’s BI service system, a preliminary investigation was undertaken. This endeavor involved conducting interviews with pertinent stakeholders intimately involved in service provision. The selection criteria for these stakeholders emphasized substantial experience and affiliation with support systems, rendering their insights particularly valuable. The objective of the preliminary study was to comprehensively delve into the system’s challenges and intricacies. Key stakeholders, including tobacco control experts, health providers, and officials from various administrative levels, were consulted to identify challenges in the current BI service system. The preliminary study identified two problems underlying low effectiveness: Inadequate training for BI providers resulting in a poor quality of BI services, and no effective strategy for improving access to these services. The researchers quantified the human, financial, and material resources, and macro-management support, that would be required at the national, regional, and provincial levels to implement a hypothetical BI strategy to increase smoker quit rates.

## 2 Materials and methods

In this study, we assessed the cost-effectiveness of candidate BI strategies for the current BI system in Thailand. The research focused on the “O” component of MPOWER, which reflects the policy of the Thai Ministry of Public Health (MOPH). The main health outcomes used to estimate BI effectiveness were smoking prevalence, the number of NCD-related deaths averted, and the number of Healthy Life-years (HLY) gained. The main program and intervention costs required for the implementation of each BI strategy were calculated. Program costs consist of human resources, as well as training, supervision, monitoring, evaluation, advocacy, infrastructure, general equipment, and overhead management costs. Intervention costs consist of the cost for healthcare to help smokers quit and BI specific supplies. Cost-effectiveness analysis of the different strategies was conducted using the incremental cost-effectiveness ratio (ICER) indicator (19).

### 2.1 Data collection and management

Acknowledging the paucity of conclusive evidence regarding the effect size of BI, our endeavor to enhance tobacco cessation BI strategies and ascertain their cost-effectiveness embraced a meticulously crafted data collection and management protocol, spanning four distinct phases: Exploration, strategy development, verification, and data analysis. This comprehensive approach involved soliciting insights from experts through a series of 15 key informant interviews. A diverse panel of experts with substantial experience in BI service systems was chosen to offer insights. These experts, drawn from varied service provision backgrounds, contributed valuable perspectives based on their deep understanding of analogous contexts. By amalgamating insights from multiple experts and conducting sensitivity analyses, the credibility of projections was significantly enhanced. While empirical data will eventually be collected, expert opinion plays a pivotal role in guiding the initial exploration and identifying potential effects. This iterative approach, combining expert insights with empirical evidence, results in a more nuanced and sophisticated analysis. The data collection phases are as follows.

In the exploration phase the current BI service system (i.e., the “status quo”) was investigated. Target data for exploration included the BI service system’s main activities, assumptions on quantity of interventions required, and calculation of the unit price for each essential activity. An example of this would be for a BI provider who conducts four sessions of ten-minute interventions for each smoker, with a unit price of US$3 per smoker. Conducting the ten-minute BI service is the main activity, and a series of four BI sessions is the quantity assumption. In addition, a cost of $3 per smoker receiving this BI service is a unit price, which is calculated from the salary for 40-min equivalent of the nurse who providing the service.

In the strategy development phase, the researchers constructed hypothetical strategies for improving the BI service delivery system from the data obtained in the exploration phase. This study did not aim to design an alternative BI service *per se* but, rather, explored system improvements in order to more systematically deliver the internationally-recommended BI service.

In the verification phase, the main activities, quantity assumptions, and unit prices of each BI-improvement strategy were validated by the key informants.

During the final phase, encompassing data analysis, the researchers employed the OneHealth Tool (OHT) software. The OHT is specifically designed to facilitate strategic health planning, and was developed by the UN Inter-Agency Working Group. OHT is an indispensable resource for quantifying costing and technical support as part of the evaluation of the financial implications and health outcomes of interventions of interest. The costing framework encompasses personnel, training, monitoring/evaluation, and infrastructural overheads (e.g., water and electricity costs), formulated to project both cost dynamics and health impacts, inclusive of population growth, mortality reduction, and the diminishment of disease incidence or prevalence as intervention coverage expands (20–24). In the case of the present study, the indicators encompass rates of successful smoking cessation, averting pre-mature death, and the accrual of healthy life years (HLY).

### 2.2 Strategies to improve BI for smoking cessation

Based on the data collected from the exploration phase, we hypothesize four strategies to improve BI for smoking cessation. Each scenario delivers the BI service based on its capacity for the number of Thai smokers in 2021. For all four scenarios, the population of interest is Thais age 15 years or older residing in Thailand at the time of the study. The population in need of BI service are current smokers who reported smoking either regularly or occasionally within the past 12 months (i.e., 17.4% of the population age 15 years or older) (17).

We selected four BI-service system improvements based on two system-intervention approaches: Increasing BI service quality, and enhancing BI service accessibility. See Table 1 for details of the strategy descriptions, and their coverage rates and effectiveness.

**Table 1:** Four BI strategies and hypothesized coverage and effectiveness rates.

Strategy	Acronym	Strategy description		BI coverage	BI effectiveness
Strategy 1	Status quo: current-BI (C-BI)	In the current situation in Thailand, 15.6% of smokers had access to cessation services, and 3.7% were in the process of quitting tobacco. Tambon Health Promoting Hospitals (THPH), district and provincial hospitals comprise only 3.5% of all MOPH health service facilities in Thailand. The providers have uneven capacity to deliver the BI, and most need to acquire more confidence in providing the BI, ensuring 30 min of tobacco cessation BI session.		15.6%	3.7%
Strategy 2	Effective-training standard-BI (ET-BI)	- Standard BI service (5 min per BI session); plus - New training package and systematic management		15.6%	7.4%
Strategy 3	Current-BI plus village health volunteers (VHV) mobilization (C-BI + VHV)	- Current BI service, and ineffective training and non-systematic management as in Strategy 1 plus - VHV mobilization		33.2%	3.7%
Strategy 4	Effective-training BI plus the VHV mobilization (ET-BI + VHV)	- Standard BI service, new training package and systematic management as in Strategy 2 plus - VHV mobilization		33.2%	7.4%

The four strategies are arrayed as follows: Strategy 1: The status quo (no change in the approach that is currently being used by MOPH providers); Strategy 2: Implementing an effective training module to improve providers’ quality in BI service; Strategy 3: Mobilizing community-based village health volunteers (VHV) to increase the accessibility to BI service for smokers; and Strategy 4: A combination of Strategies 2 and 3.

Strategy 1 represents the status quo (hereafter denoted as ‘C-BI’). The C-BI service is offered in primary care settings nationwide, but is not systematically managed because only 3.5% of health care providers in the country have been trained to deliver the BI. Based on our preliminary assessment, five health personnel per province had received BI training, resulting in 385 trained people from 77 provinces, accounting for 3.5% of 10,666 primary healthcare providers in Thailand. The MOPH claimed that all of its healthcare providers offered the C-BI service, but many facilities did not have trained providers (25). Furthermore, the prevailing training package consists of only a two-day training workshop without any follow-up supervision. Therefore, many healthcare providers took an inordinate amount of time to offer the service and, thus, C-BI was not optimal or cost-effective. Based on the preliminary assessment, the providers spent 30 min per session talking about multiple topics instead of the required 5 min per session talking specifically about smoking cessation motivation. Hence, we defined Strategy 1 as a status quo situation providing the current, ineffective BI service, with ineffective training and non-systematic management. As the ‘status quo’ situation, providers verbally screen their adolescent and adult catchment population for smoking on an annual basis. Persons who admit to being smokers are offered help to quit tobacco use. This strategy’s BI service covers 15.6% of Thai smokers and its effectiveness for quitting smoking is 3.7%.

Strategy 2 improves the BI service system by providing effective training for the BI providers in all primary healthcare settings to deliver standard BI service. Its acronym is ‘ET-BI’ which stands for effective training BI. In Strategy 2, the MOPH would offer a new BI training package that includes a two-day training workshop followed by two supervision sessions. The two-day training workshop would address the standard BI practice for smoking cessation (i.e., providing four, 5-min BI sessions per smoker). Trained BI providers would apply their real-world experience with the BI practices to consult with the trainers during two supervision sessions after the training to correct any errors. The MOPH would also train the BI providers working in all primary healthcare settings nationwide. During our verification phase, field experts confirmed that this approach could double the effectiveness of Strategy 1, from 3.7 to 7.4%.

Strategy 3 improves the BI service system by increasing smokers’ access to BI over the C-BI service by mobilizing cadres of VHV to visit smokers in the community and encourage them to receive BI at the nearby primary healthcare provider. Its acronym is ‘C-BI + VHV’ standing for the current BI system plus the VHV approach. The primary healthcare system in Thailand consists of 9,766 sub-district health centers and 882,226 VHV (26). This is equivalent to 90 VHV per sub-district health center, and one VHV is assigned to take care of 10–15 households. In Strategy 3, the MOPH would mobilize 882,226 VHV nationwide to encourage smokers to seek BI service. Typically, VHV meet with the director of the local sub-district health center for 3 h every month to update their knowledge regarding community healthcare. In our Strategy 3, the VHV would be trained by the director of the sub-district health center to build skills in motivating smokers to use the BI service. The director would use video clips of best practices to train their cadre of VHV in a one-hour session, three times per year. The trained VHV would be expected to visit 15 smokers at least three times per year (if needed) to urge them to seek the local BI service. Based on our verification, field experts agreed that this VHV mobilization approach could double the BI accessibility rate from 15.6 to 33.2%.

Strategy 4 is a combination of Strategies 2 and 3: Effectively training BI providers to deliver the standard BI service (Strategy 2), plus mobilizing VHV to screen and encourage smokers to seek BI service (Strategy 3). We hypothesize that Strategy 4 will have a smoking cessation success rate of 7.4% (similar to Strategy 2) and an accessibility rate of 33.2% (similar to Strategy 3).

For the purpose of this research, all four strategies were defined as nine-year interventions, to occur between 2022 and 2030. The first 3 years were designated as the preparation phase, which involves BI provider capacity building. During the six-year implementation phase, all strategies are applied at maximum capacity. In Strategies 2, 3, and 4, the target coverage rates for the first 3 years are defined as a progressive coverage increase from 50 to 80 to 100%, respectively. For Years 4 to 10, coverage is held constant at 100%. The effectiveness of each strategy varies depending on the quality of the capacity building, with Strategies 2 and 4 contributing a 7.4% effectiveness that is twice as large as Strategies 1 and 3 (3.7%). Table 2 shows a summary of the hypothesized coverage rates and effectiveness defined for each of the four BI strategies during the nine-year prediction interval.

**Table 2:** Summary of BI coverage and effectiveness by strategy by year.

Strategy	Coverage	Intervention effectiveness (IE)		2022 (50%)	2023 (80%)	2024 (100%)	2025	2026	2027	2028	2029	2030
Strategy 1	15.6%	3.7%	Coverage	15.6%	15.6%	15.6%	15.6%	15.6%	15.6%	15.6%	15.6%	15.6%
			Impact (IE)	3.7%	3.7%	3.7%	3.7%	3.7%	3.7%	3.7%	3.7%	3.7%
Strategy 2	15.6%	7.4%	Coverage	15.6%	15.6%	15.6%	15.6%	15.6%	15.6%	15.6%	15.6%	15.6%
			Impact (IE)	7.4%	7.4%	7.4%	7.4%	7.4%	7.4%	7.4%	7.4%	7.4%
Strategy 3	33.2%	3.7%	Coverage	24.4%	30.0%	33.2%	33.2%	33.2%	33.2%	33.2%	33.2%	33.2%
			Impact (IE)	3.7%	3.7%	3.7%	3.7%	3.7%	3.7%	3.7%	3.7%	3.7%
Strategy 4	33.2%	7.4%	Coverage	24.4%	30.0%	33.2%	33.2%	33.2%	33.2%	33.2%	33.2%	33.2%
			Impact (IE)	7.4%	7.4%	7.4%	7.4%	7.4%	7.4%	7.4%	7.4%	7.4%

### 2.3 Outcome measures

Outcome indicators of effectiveness of the four BI strategies include the prevalence of smoking, the number of NCD-related deaths averted, and the number of HLY gained. To calculate these outcomes, we employed the OHT parameters and estimation capacity based on a comprehensive literature review and meta-analysis (27–29) carried out by the OHT team at WHO. Hence, we use the default values of the OHT software, and that can be considered a limitation of this study. However, we updated the values of the prevalence rate based on the latest round (2021) of the Thai national Health Behavior of the Population survey. The parameters include total population, current smoking prevalence by age and sex, global-average relative risks of smoking for various NCD by age and sex (including ischemic heart disease, stroke, diabetes mellitus, asthma, chronic obstructive pulmonary disease, and cervical cancer), and attributable fractions (e.g., ischemic heart disease, stroke, diabetes mellitus, and chronic obstructive pulmonary disease). The OHT analysis providers an estimation of the strategy effectiveness by key parameters, relative risks, and attributable fraction in the population.

We entered the required data into the OHT software for analysis and projection of the effectiveness of the BI to quit tobacco use according to the outcome indicators. OHT provides the estimates of the gender- and age-specific prevalence of smoking (based on secular trends minus the impact of the intervention), the resulting numbers of NCD-related deaths averted by sex (assuming no other relevant interventions impacting mortality), and the number of HLY gained annually throughout the study period (2022-30).

### 2.4 Cost estimation of the interventions

The cost estimation for the BI system-improvement intervention was derived from the costs of the BI specific activities (i.e., the intervention cost in the OHT) and BI support activities (i.e., the program cost in the OHT). The program and intervention costs were the primary components of the total cost estimates. The program costs consist of the costs for human resources, training, supervision, monitoring, evaluation, advocacy, infrastructure, general equipment, and management. The intervention costs include the provider’s time used to deliver the BI, and BI supplies (e.g., paper and pen). We collected the cost data at macro and micro levels. Macro costing data were obtained from the Chairperson of the Division of Tobacco Product Control Committee, representatives from two MOPH regional offices for disease prevention and control, and representatives from two provincial health offices (PHO). The micro costing data were collected from healthcare service representatives from three provincial hospitals, representatives from three district hospitals, and representatives from three THPH.

The data in Supplementary Table S1 define the main BI activity.

Each year of Strategy 1 (status quo) includes ten core activities comprising the following: (1) Two meetings of the National Tobacco Control Board; (2) Eight meetings among staff of the Tobacco Control Division (under the Department of Disease Control, MOPH); (3) One policy instruction meeting between the Tobacco Control Division and PHO from all 77 provinces of Thailand; (4) A provincial policy cascade meeting among relevant health officers within each of the 77 provinces; (5) A three-day training of trainers (only for the first year); (6) A two-day BI training; (7) Two routine supervision sessions; (8) Print media (e.g., guidebook, infographics, posters, brochures); (9) Two monitoring and evaluation meetings between the Tobacco Control Division and the provincial level; and (10) Two monitoring and evaluation meetings for relevant health personnel within each province. Strategy 1 contains four 30-min BI sessions.

Strategy 2 has the same activities as Strategy 1, except that its BI training is a two-day workshop, two BI practice supervision sessions, and in-service education through an online learning platform for BI providers. Furthermore, its BI service contains four, 5-min BI sessions.

Strategy 3 has the same activities as Strategy 1, except that it has additional activities for VHV capacity-building, which includes three, 1-h learning sessions using five video clips, and learning through an online platform for VHV.

Strategy 4 combines the activities found in Strategies 2 and 3. The cost of VHV mobilization consists of function-related costs, e.g., training and annual meeting.

The calculation of indirect costs in all strategies involved the accounting of meal and transportation allowances for smokers. However, in Strategies 3 and 4, the indirect costs associated with Village Health Volunteers (VHV), such as labor and opportunity costs, were not factored into the cost calculation. This exclusion was justified by the assumption that the BI was integrated into routine VHV activities.

The questionnaires were systematically developed in accordance with the health costing frameworks established by the WHO within the OHT software (20). Unit prices for essential activities within the BI service domain were acquired through an expressly designed questionnaire survey. This survey was administered to 43 individuals directly involved in BI service activities, representing diverse settings and hierarchical levels within the healthcare system. These key informants include a person from the national level, three people from regional level, three people from the provincial level, four people from tertiary care hospitals, six people from secondary care hospitals, and 26 people from the primary care level. There are too many details regarding the main unit price calculation to describe fully in this article. The quantity assumptions and the unit prices of each core activity were input to calculate the unit cost values and, thereby, the intervention and program cost estimates (OHT 2019, when approximately 34 baht equaled one US dollar).

### 2.5 Cost-effectiveness analysis

We employed Incremental Cost-effectiveness Ratio (ICER), a fundamental metric in health economics used to compare the relative value of different healthcare interventions, to compare the cost-effectiveness of the four BI strategies (30). The calculation involved finding the cost and effectiveness differences between the interventions, then dividing the cost difference by the effectiveness difference. This yields the ICER, representing the additional cost incurred for gaining one additional unit of effectiveness. Decision-makers use ICER to evaluate the cost-effectiveness of interventions (by comparing the ratio to a predetermined threshold) in order to inform healthcare resource allocation decisions. In this study, we calculated the ICER in terms of US dollars per smoking quitter, NCD-related deaths averted, and per HLY gained for Strategies 2, 3, and 4 by using Strategy 1 as reference (Supplementary Table S2). Moreover, in order to quantify uncertainty, optimize decisions under diverse scenarios, and augment model credibility, a sensitivity analysis involving nine distinct strategies was conducted. The results of this analysis indicated no statistically-significant differences in effectiveness among the strategies examined (Supplementary Table S3). As a result, our study primarily focused on presenting four main strategies in its findings.

### 2.6 Ethical approval

The data collection tool and procedures complied with local and national regulations. Participants were informed of the purpose of the study. The study protocol was approved by the ethical committee of Institute for Population and Social Research, Mahidol University number; IPSR-IRB-2021-075. Service clients and the public WERE NOT involved in the design, or conduct, or reporting, or dissemination plans of our research.

## 3 Results

Table 3 provides a summary of the impacts of the four, nine-year BI-improvement interventions with respect to four health outcomes: Smoking prevalence, cumulative number of smoking quitters, cumulative number of NCD-related deaths averted, and the cumulative number of HLY gained. After the theoretical nine-year period of implementation, Strategies 2, 3 and, 4 provided better results than Strategy 1, with Strategy 4 performing the best. In Strategy 4, the smoking prevalence in 2030 is 16.6%, with an −0.8% absolute reduction in prevalence compared to 2021, when it was 17.6%. Over the 9 years (from 2022 to 2023) of observation, the cumulative number of smoking quitters, NCD-related deaths averted, and HLY gained were 251,108, 2,722, and 25,591, respectively.

**Table 3:** Projection effectiveness of BI strategy for tobacco cessation in Thailand.

Parameters	System-improvement intervention strategies			
	Strategy 1	Strategy 2	Strategy 3	Strategy 4
1. Smoking prevalence rate in 2030 (Prevalence in 2021 = 17.4%)	16.9%	16.8%	16.8%	16.6%
Males (prevalence at 2021 = 34,7%)	33.7%	33.5%	33.5%	33.0%
Females (prevalence at 2021 = 1.35%)	1.4%	1.4%	1.4%	1.4%
2. Absolute smoking prevalence change between 2021 and 2030	−0.5%	−0.6%	−0.6%	−0.8%
Males	−1.0%	−1.2%	−1.2%	−1.7%
Females	0.0%	0.0%	0.0%	0.0%
3. Cumulative number of smoking quitters	59,078	117,127	124,520	251,108
Males	56,480	112,515	119,933	241,147
Females	2,598	4,612	4,587	9,962
4. Cumulative number of NCD-related deaths averted	676	1,351	1,354	2,722
Males	544	1,089	1,093	2,197
Females	132	262	261	525
5. Cumulative number of HLY gained	6,420	12,868	12,746	25,591
Males	5,180	10,387	10,288	20,668
Females	1,240	2,481	2,458	4,923

Table 4 summarizes the results of the nine-year cost estimations for all four BI improvement strategies. Costs are presented in US dollars, and the discount rate, inflation, and depreciation rates were set at 0.0% in all periods due to unpredictable economic fluctuation during the Thai COVID-19 epidemic. The total costs were $64,065,591, $20,447,330, $129,497,192, and $40,191,330 for Strategies 1, 2, 3, and 4, respectively. Strategies 1 and 2 could each provide the BI service to 14,582,994 smokers because they applied the same ‘business-as-usual’ service accessibility strategy. Compared to Strategy 1, Strategy 2 has better health provider training, resulting in better BI service performance, but not better accessibility. Strategies 3 and 4 each have 29,753,800 smokers receiving the BI service because both employ the same VHV accessibility-enhancing strategy. From the data in Table 4, it can be seen that the intervention cost per smoker receiving BI service is equal between Strategies 1 and 3, as well as between Strategies 2 and 4. The reason for this is that the former pair applies the same business-as-usual, high-cost, ineffective BI service (e.g., 15 min per BI session), while the latter pair employs better training, and better BI performance (e.g., international standard 5 min per BI session). The program costs per smoker receiving BI service are $0.10, 0.12, 0.06, and 0.07, for strategies 1-4, respectively. As a result, the strategies with the lowest and highest costs-per-smoker receiving BI service are strategies 4, 2, 3, and 1, with $1.35, 1.40, 4.35, and 4.39, respectively. Based on the cost-per smoking-quitter provided by each strategy (Table 4), Strategy 4 has the cheapest unit cost ($160 per smoking quitter) and cost per NCD-related death averted ($14,765 per death averted).

**Table 4:** Summary of costs by BI-improvement strategy for smoking cessation in Thailand (OHT 2019, 34 baht = 1 US dollar).

Parameters	Strategy 1	Strategy 2	Strategy 3	Strategy 4
1.Total cost	64,065,591	20,447,330	129,497,192	40,191,330
1.1 Intervention costs	62,587,292	18,677,066	127,704,868	38,107,040
1.2 Program costs	1,478,299	1,770,264	1,792,324	2,084,290
2. Number of smokers receiving BI service	14,582,994	14,582,994	29,753,800	29,753,800
3. Total cost-per-smoker receiving BI service	4.39	1.40	4.35	1.35
3.1 Intervention cost-per-smoker receiving BI service	4.29	1.28	4.29	1.28
3.2 Program cost-per-smoker receiving BI service	0.10	0.12	0.06	0.07
4. Total cost-per-smoking quitter	1,084.42	174.57	1,039.97	160.06
4.1 Intervention cost-per-smoking quitter	1,059.40	159.46	1,025.58	151.76
4.2 Program cost-per-smoking quitter	25.02	15.11	14.39	8.30
5. Total cost per NCD-related death averted	94,772	15,135	95,640	14,765

Discount, inflation, and depreciation rates were identified constantly at 0.0% during all projections due to unpredictable economic fluctuation during the Thai COVID-19 epidemic (31).

Table 5 presents a summary of the estimated ICER per smoking quitter, NCD-related deaths averted, and HLY gained, for the four BI enhancement strategies. The study assumed periods of projection during the COVID-19 epidemic, and the discount, inflation, and depreciation rates were set at 0.0%. Based on the ICER, Strategies 2 and 4 cost less per additional unit of health outcomes compared to Strategy 1. Among the strategies for improving the BI system, Strategy 2 is the most efficient compared to the status quo (Strategy 1). That approach would save $751 per additional smoking quitter, $64,620 per additional death averted, and $6,765 per HLY gained when compared to the status quo. In addition, Strategy 4 would save $124 per additional smoking quitter, $11,669 per additional death averted, and $1, 245 per HLY gained when compared to the status quo.

**Table 5:** Summary of estimated ICER per smoking quitters, NCD-related deaths averted, and HLY gained by BI-improvement strategy (OHT 2019, 34 baht = 1 US dollar).

Outcome	Strategy 1 = status quo (Comparator)		Strategy 2			Strategy 3			Strategy 4		
	Total cost	Total effects	Total cost	Total effects	ICER	Total cost	Total effects	ICER	Total cost	Total effects	ICER
Smoking quitter	64,065,591	59,078	20,447,330	117,127	−751	129,497,192	124,520	1,000	40,191,330	251,108	−124
Death averted	64,065,591	676	20,447,330	1,351	−64,620	129,497,192	1,354	96,507	40,191,330	2,722	−11,669
HLY gained	64,065,591	6,420	20,447,330	12,868	−6,765	129,497,192	12,746	10,343	40,191,330	25,591	−1,245

## 4 Discussion

In order to provide MOPH policymakers with well-informed insights conducive to enhancing the cost-effectiveness of the BI service system, the present study undertook an analytical endeavor aimed at evaluating the postulated cost-effectiveness of four distinct BI strategies, delineated along two critical dimensions: augmentation of BI service quality and enhancement of BI service accessibility. The assessments encompassed perspectives from experienced consultants, alongside an examination of the outcomes associated with BI, notably within the context of smoking cessation initiatives. The findings provide compelling evidence for the potential of initiating substantial reforms in Thailand’s tobacco-related BI strategies. The calculated Incremental Cost-Effectiveness Ratio (ICER) reveals that Strategy 2, characterized by effective training in BI service, emerges as the most economically viable approach for enhancing the BI service system when compared to the status quo (Strategy 1). The estimated total cost-per-smoking-quitter was approximately $175. This level of cost-effectiveness is lower than international benchmarks. For instance, in the United Kingdom, the cost per year of life saved through brief advice was £248 ($410), as reported in a prior study (32). Furthermore, another investigation indicated a cost of €354 per year of life saved ($495), derived from practitioners’ brief advice within smoking cessation services. These comparative metrics underscore the significance of Strategy 2 as a financially prudent and impactful means of advancing tobacco-related intervention efforts (33). Note that all strategies are examined relative to Strategy 1, where the accessibility of the current strategy is already factored in. Strategy 2 is the most cost-effective strategy for two reasons. First, Strategy 2 has a lower BI service cost because it provides four, 5-min BI sessions for each smoker instead of four 30-min BI sessions (as provided in the status quo strategy). In this strategy, the BI smoking cessation program was hypothetically provided to 60 smokers age 15 years or older. One-third (20 smokers) were projected to have quit smoking after 6 months of the program. This approach has been shown to be effective in helping smokers stay tobacco-free at 1 month (42.7% quitting rate) and at 6 months (35.0% quitting rate) after receiving the full BI service (34). Secondly, Strategy 2 has better BI training with the provision of two BI practice supervision sessions after the two-day BI training workshop and online learning platform. We modeled Strategy 2 based on a study that involved 626 senior nursing students. That study found that 91.7% of the participants felt that the training program enhanced their ability to assist BI clients to quit smoking. Well-designed 5A (e.g., Ask, Advise, Assess, Assist, Arrange) training courses could increase knowledge and self-efficacy of tobacco cessation among trained nurses (35). The government costs for improving BI service according to strategy 2 are around $20,447,330 for the 9 years, which is less than the cost of Strategy 1. Moreover, the online educational program and learning through BI practice were able to increase trainees’ confidence and smoking cessation counseling skills. Students self-assessed higher levels of smoking cessation skills on Advising, Assessing, Assisting, and Arranging compared to baseline (36). Hence, the government costs for the improved BI service as per Strategy 4 are projected to be around $40,191,330 for the 9 years, which is less than Strategy 1.

The reason that Strategy 4 is the most effective, but not the most efficient, strategy, is because Strategy 4 broadens the accessibility of smoking cessation together with improving training, thereby incurring additional costs. The increased accessibility of Strategy 4 is based on Strategy 3 (the VHV- mobilization approach). Thailand has a long history of VHV involvement in assisting health personnel in service provision going back to the 1960s (37). Successful examples of VHV involvement in healthcare service provision include improvement in the nutritional status of children under the age of 5 years, household access to clean water, immunization coverage, expanded availability of essential drugs, as well as suicide prevention (38). There is evidence that VHV can be trained to assist smoking cessation in Thailand as well. A Thai quasi-experimental study in 2019 assessed the level of smoking-cessation-assistance ability among 64 VHV before and after participating in a program to boost health literacy for smoking cessation. That study found that the competency score after participating in the training program was significantly higher than the baseline score (*t* = 2.78, value of *p* < 0.5) (39). Thus, Strategy 4 is the best choice for achieving the global target of reducing smoking prevalence in Thailand through VHV training and involvement in smoking cessation provision. That said, Strategy 4 is less efficient in achieving additional quitters, NCD-related deaths averted, and HLY gained.

In sum, based on our findings, if the MOPH wishes to increase the effectiveness of the national BI-service system in the most cost-effective manner, they should adopt Strategy 2, which is an enhanced BI service system using effective training for primary healthcare personnel. If the government has the budget available, it could adopt Strategy 4, which is the most effective strategy for improving the BI service performance through the use of the effective BI training program and by involving VHV to increase accessibility to BI service.

Before integrating the study’s findings into practical applications, it is crucial to address inherent limitations. One significant concern is the scarcity of research evidence, particularly regarding effect size, which necessitates careful interpretation. These limitations encompass various aspects, including the methods employed for data collection, expert opinions sought, and the choice of discount rate. Notably, data collection was confined to the perspective of the MOPH, emphasizing its specific role in BI services. Additionally, the evaluation of intervention costs considered only direct expenses, thus omitting crucial indirect costs from the analysis. Insights into service delivery heavily relied on estimates provided by healthcare professionals, which were validated by domain experts. Although the uniformity of discount and inflation rates employed in the analysis offers methodological convenience, it may not comprehensively capture the complexities of real-world dynamics. Therefore, the integration of the study’s findings into practical contexts should be accompanied by a nuanced consideration of these limitations, ensuring a mindful and contextually-appropriate interpretation.

## 5 Conclusion

This study provides evidence that improvements to the national BI service system would be most cost-effective if Strategy 2 is applied (among four hypothetical scenarios). The study has demonstrated that the prevalence of tobacco use in Thailand could be reduce further by enhancing training and expanding access to BI for more smokers. The suggested changes to the current system for delivering BI services, as suggested in this study, could contribute to more efficient and effective health outcomes related to smoking cessation. Service delivery, access, and administration should be widened, and more effective service training should be incorporated.

## Data availability statement

The original contributions presented in the study are included in the article/Supplementary material, further inquiries can be directed to the corresponding author.

## Ethics statement

This study protocol was approved by the ethical committee of Institute for Population and Social Research, Mahidol University number; IPSR-IRB-2021-075. Patients and the public were not involved in the design, or conduct, or reporting, or dissemination plans of our research.

## Author contributions

RP: Conceptualization, Data curation, Formal analysis, Investigation, Methodology, Project administration, Validation, Writing – original draft, Writing – review & editing. BS: Conceptualization, Formal analysis, Methodology, Supervision, Writing – original draft, Writing – review & editing. YS: Conceptualization, Supervision, Validation, Writing – review & editing. SC: Conceptualization, Formal analysis, Supervision, Validation, Writing – review & editing. PS: Conceptualization, Formal analysis, Funding acquisition, Supervision, Writing – review & editing. PK: Conceptualization, Data curation, Formal analysis, Investigation, Methodology, Project administration, Supervision, Validation, Writing – original draft, Writing – review & editing. JR: Conceptualization, Formal analysis, Methodology, Project administration, Supervision, Writing – original draft, Writing – review & editing.
