# Supplementary material for: The cost-effectiveness of improved brief interventions for tobacco cessation in Thailand
Source: Front Public Health. 2023 Nov 23;11:1289561. doi: 10.3389/fpubh.2023.1289561 (PMC10701387; doi:10.3389/fpubh.2023.1289561)
Supplement: Supplementary file 1 [file Data_Sheet_1.PDF]

TABLE S1: The main activity and quantity assumptions for all four of strategies

Main Activity	Strategy 1	Strategy 2	Strategy 3	Strategy 4
**BI-supporting activities**				
1. National Tobacco Control Board meeting	twice per year	twice per year	twice per year	twice per year
2. Meeting among staffs of the Tobacco Control Division	8 times/ year	8 times/ year	8 times/ year	8 times/ year
3. Policy-instruction meeting	once per year	once per year for provincial head	Once per year for provincial head	Once per year for provincial head
			Once per year for health service support	Once per year for health service support
4. Provincial policy-cascade meeting	Once per year	Once per year	Once per year	Once per year
5. Training of the Trainer	A 3-day workshop (only one time in the first year of preparation phase)	A 3-day workshop (only one time in the first year of preparation phase)	A 3-day workshop (only one time in the first year of preparation phase)	A 3-day workshop (only one time in the first year of preparation phase)
			-Training for VHV trainer 1 day	-Training for VHV trainer 1 day
6. BI training	A 2-day workshop	A 2-day workshop plus two BI-practice supervision sessions	A 2-day workshop	A 2-day workshop plus two BI-practice supervision sessions
7. Operation supervision	twice	twice	twice	twice

Main Activity	Strategy 1	Strategy 2	Strategy 3	Strategy 4
8. Mass media	Guidebook, Infographic, Poster, Brochure	Guidebook, Infographic, Poster, Brochure	Guidebook, Infographic, Poster, Brochure	Guidebook, Infographic, Poster, Brochure
9. Monitoring and evaluation by provincial level to provincial level	twice per year	twice per year	twice per year	twice per year
10. Monitoring and Evaluation by provincial level to health personal	twice per year	twice per year	twice per year	twice per year
11. online learning		set up and operate an online learning platform for the BI providers	set up and operate an online learning platform for the VHVs	set up and operate an online learning platform for the BI providers<br>set up and operate an online learning platform for the VHVs
12. Training for VHVs			3 one-hour learning sessions, using 5 video clips	3 one-hour learning sessions, using 5 video clips
**BI-specific activities**				
14. BI service: intervention costs included the cost per smoker receiving the BI service and the cost of supplies.	Four 30-minute BI sessions	Four 5-minute BI sessions	Four 30-minute BI sessions	Four 5-minute BI sessions

Note: boxes highlighted in lighter gray are activities that Models 2 and 4 have but Model 1 does not, while boxes highlighted in darker gray are activities that Models 3 and 4 have but Model 1 does not.
