# Supplementary material for: The cost-effectiveness of improved brief interventions for tobacco cessation in Thailand
Source: Front Public Health. 2023 Nov 23;11:1289561. doi: 10.3389/fpubh.2023.1289561 (PMC10701387; doi:10.3389/fpubh.2023.1289561)
Supplement: Supplementary file 2 [file Data_Sheet_2.PDF]

TABLE S2: A summary of the strategy for improved brief interventions for tobacco cessation in Thailand based on projection

Parameters	Strategies			
	**Strategy 1**	**Strategy 2**	**Strategy 3**	**Strategy 4**
**Total cost**	64,065,591	20,447,330	129,497,192	40,191,330
**Quitter**				
- Effectiveness	59,078	117,127	124,520	251,108
-CEA	1,084	175	1,040	160
**Death Averted**				
- Effectiveness	676	1,351	1,354	2,722
-CEA	94,772	15,135	95,640	14,765
**HLY gained**				
- Effectiveness	6,420	12,868	12,746	25,591
-CEA	9,979	1,589	10,160	1,571
