# Supplementary material for: The cost-effectiveness of improved brief interventions for tobacco cessation in Thailand
Source: Front Public Health. 2023 Nov 23;11:1289561. doi: 10.3389/fpubh.2023.1289561 (PMC10701387; doi:10.3389/fpubh.2023.1289561)
Supplement: Supplementary file 3 [file Data_Sheet_3.PDF]

A summary of sensitivity of effectiveness of the all strategies for tobacco cessation in Thailand based on projection

Strategies	Parameters	
	Death Averted	HYL gained
Strategy 1 (15.6%, 3.7%)	676	6,420
Strategy 2 (15.6%, 5.4%)	984	9,361
Strategy 3 (15.6%, 7.4%)	1,351	12,868
Strategy 4 (24.4%, 3.7%)	1,016	9,576
Strategy 7 (33.2%, 3.7%)	1,354	12,746
Strategy 5 (24.4%, 5.4%)	1,480	13,979
Strategy 8 (33.2%, 5.4%)	1,980	18,599
Strategy 6 (24.4%, 7.4%)	2041	19,259
Strategy 9 (33.2%, 7.4%)	2,722	25,591
